# Automated Brain Masking of Fetal Functional MRI with Open Data

**DOI:** 10.1007/s12021-021-09528-5

**Published:** 2021-06-15

**Authors:** Saige Rutherford, Pascal Sturmfels, Mike Angstadt, Jasmine Hect, Jenna Wiens, Marion I. van den Heuvel, Dustin Scheinost, Chandra Sripada, Moriah Thomason

**Affiliations:** 1 Donders Institute, Radboud University Medical Center, Nijmegen, The Netherlands; 2 Department of Psychiatry, University of Michigan, MI Ann Arbor, USA; 3 Department of Electrical Engineering and Computer Science, University of Michigan, Ann Arbor, MI, USA; 4 Department of Psychology, Wayne State University, Detroit, MI, USA; 5 Department of Cognitive Neuropsychology, University of Tilburg, Tilburg, The Netherlands; 6 Department of Radiology and Biomedical Imaging, Yale School of Medicine, New Haven, CT, USA; 7 Department of Statistics and Data Science, Yale University, New Haven, CT, USA; 8 Child Study Center, Yale School of Medicine, New Haven, CT, USA; 9 Department of Child and Adolescent Psychiatry, New York University School of Medicine, New York, NY, USA; 10 Department of Population Health, New York University School of Medicine, New York, NY, USA

**Keywords:** Fetal, fMRI, Functional imaging, Brain segmentation, Deep learning, Convolutional neural network, Open-source software

## Abstract

Fetal resting-state functional magnetic resonance imaging (rs-fMRI) has emerged as a critical new approach for characterizing brain development before birth. Despite the rapid and widespread growth of this approach, at present, we lack neuroimaging processing pipelines suited to address the unique challenges inherent in this data type. Here, we solve the most challenging processing step, rapid and accurate isolation of the fetal brain from surrounding tissue across thousands of non-stationary 3D brain volumes. Leveraging our library of 1,241 manually traced fetal fMRI images from 207 fetuses, we trained a Convolutional Neural Network (CNN) that achieved excellent performance across two held-out test sets from separate scanners and populations. Furthermore, we unite the auto-masking model with additional fMRI preprocessing steps from existing software and provide insight into our adaptation of each step. This work represents an initial advancement towards a fully comprehensive, open-source workflow, with openly shared code and data, for fetal functional MRI data preprocessing.

## Introduction

Resting-state functional magnetic resonance imaging (rs-fMRI) has emerged as a powerful tool for studying the brain’s network architecture development. In recent years, this methodology has been applied to the human brain *in utero*, opening a window into a functional growth period that was otherwise inaccessible. Studying fetal fMRI has the potential to illuminate the nature and manner in which the brain’s network architecture is initially assembled, affording powerful new insights into neurodevelopmental origins (Jakab et al., 2014, 2015; Schöpf et al., 2012; Thomason et al., 2017; van den Heuvel et al., 2018). Despite this potential, progress has been slow due, in part, to the lack of image analysis tools tailored for fetal imaging data. Though many tools and software packages exist for fMRI analyses, these tools were designed with adult and child data in mind and encounter specific problems when applied to fetal functional data.

Progress made towards improving fetal MRI methodology can broadly be divided into image acquisition and image post-processing. Image acquisition improvements have occurred mainly concerning fetal *structural* MRI, particularly in anatomical (primarily T2 HASTE; Half-Fourier Acquisition Single-shot Turbo spin Echo imaging) and diffusion tensor imaging (DTI) (Benkarim et al., n.d.; Biegon & Hoffmann, 2014). Advances have been made in inter-slice motion correction and volume reconstruction (Fogtmann et al., 2014; Gholipour et al., 2017; Studholme, 2011), mapping structural connectivity (Huang et al., 2018; A. Ouyang et al., 2015; M. Ouyang et al., 2018; Qiu et al., 2015; Song et al., 2017; Takahashi et al., 2012), and comparing different MRI signals (Seshamani et al., 2015). These strategies have made it possible to use sparse acquisition sequences, which alleviate movement concerns (Serag et al., 2017; Seshamani et al., 2014), and enable more sophisticated analytic approaches, such as morphometric (Gholipour et al., 2017; Kuklisova-Murgasova et al., 2011; Serag et al., 2012; Shi et al., 2010; Studholme, 2011) cortical folding (Wright et al., 2014), and cytoarchitectural examinations (Miller et al., 2014). In contrast, papers focusing on image post-processing improvements are markedly few and primarily focused on structural imaging. Articles suggesting possible solutions for fetal *functional* MRI data analysis are rare (Scheinost et al., 2018; Seshamani et al., 2014). In this work, we focus on methods for improving image post-processing for fetal functional MRI data.

Challenges associated with the analysis of fetal fMRI have been discussed in a growing number of studies (Schuler et al., 2018; Thomason, 2018; Thomason et al., 2014, 2015, 2017; van den Heuvel et al., 2018) and reviews (Anderson & Thomason, 2013; A. J. Robinson & Ederies, 2018; van den Heuvel & Thomason, 2016; Vasung et al., 2018). These works have focused on image characteristics: motion, size of the fetal brain, susceptibility artifacts introduced by surrounding maternal tissues, and physiological noise of both mother and fetus. Previous work has highlighted essential areas for development, but to our knowledge, no one has proposed a preprocessing pipeline for fetal functional MRI and released it in the open science framework.

The most time-consuming step in preprocessing fetal fMRI is the differentiation of the fetal brain from the surrounding maternal compartment at each acquisition time point. Differentiation is achieved by the generation of an exemplar mask that marks all in-brain voxels. This mask is critical for the entire preprocessing pipeline and subsequent activation or connectivity analyses. Tools developed to segment the adult brain, such as the Brain Extraction Tool (BET) from FSL (Jenkinson et al., 2002) and 3dSkullstrip (3dSS) from AFNI (Cox, 1996), are not effective in generating exemplar masks in fetal imaging for numerous reasons. There is surrounding maternal tissue in fetal images instead of the black background in adult brain images. The fetal brain is not in a standard orientation and shape assumptions do not hold making the atlas-based extraction priors of adult brain segmentation tools inapplicable. As a result, previous studies have relied on the manual generation of brain masks (Thomason et al., 2013, 2014, 2015, 2017; van den Heuvel et al., 2018). While manual methods are tedious and time-consuming, to date, they have been the predominant approach to achieve acceptable standards.

**Fig. 1:**
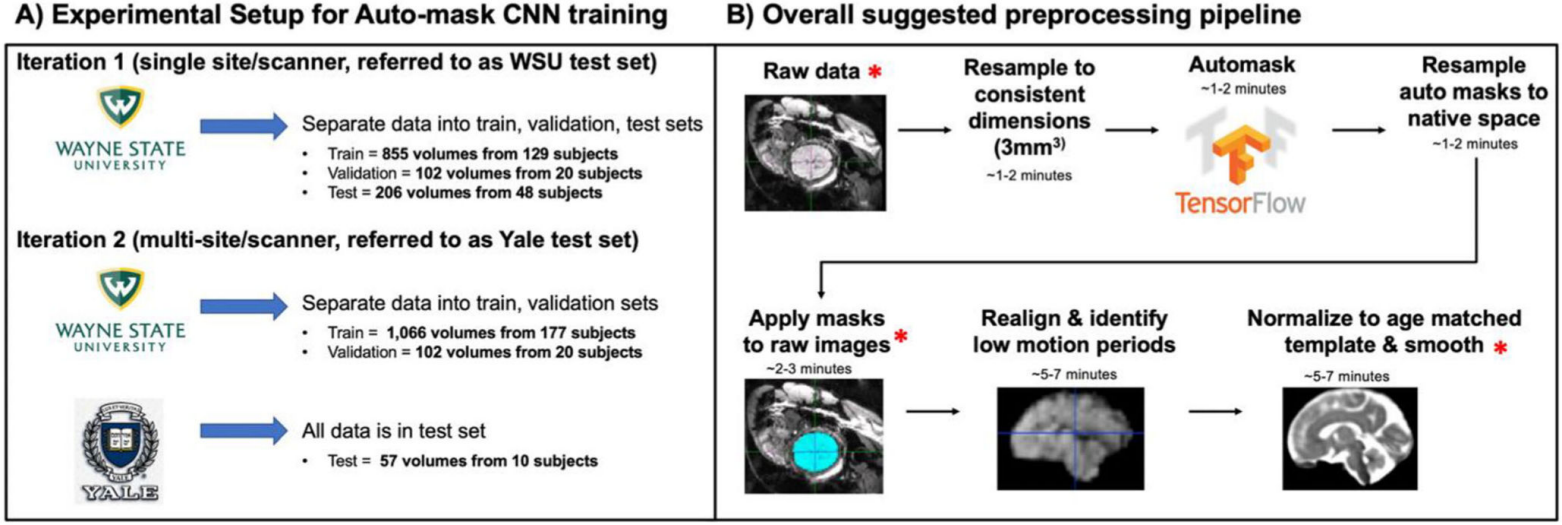
Overview of the experimental pipeline for training, validation, and testing of the convolutional neural network (CNN) auto-mask model and the proposed preprocessing pipeline. (A) Details of how data were separated into training, validation, and test sets. Two iterations of the auto-mask CNN model were run to compare single-site results (iteration 1) with multi-site results (iteration 2). (B) All steps in the proposed preprocessing stream are shown, with a red asterisk representing where visually quality checking data is recommended. This workflow can be run as shell scripts from the command line and allows for user flexibility

Here, we present an automated approach to the problem of fetal brain segmentation from surrounding tissue. Leveraging a large corpus of manually traced human fetal fMRI masks, we trained a convolutional neural network (CNN) to replace this labor-intensive preprocessing step. CNN’s are a powerful tool for effectively identifying complex, non-linear patterns in spatially structured high-dimensional datasets (Lecun et al., 1998). They are increasingly utilized in image processing applications in both medical and non-medical settings (Egmont-Petersen et al., 2002; Falk et al., 2018; Zeiler & Fergus, 2014). In the context of fetal brain segmentation, prior work has investigated the application of CNN’s to segment fetal structural T2-weighted volumes (Ebner et al., 2020; Ison et al., 2012; Khalili et al., 2019; Klinder et al., 2015; Link et al., 2017; Makropoulos et al., n.d.; Payette et al., 2021; Rajchl et al., 2016; Salehi et al., 2017, 2018; Serag et al., 2017; Tourbier et al., 2017). These models, however, were developed to segment the fetal brain from anatomical images and do not translate to functional time series data. Compared to structural data, functional data are typically lower resolution and due to movement require a larger quantity of individual segmentations. The majority of existing fetal MRI datasets that have been utilized for deep learning brain segmentation are collected in clinical settings where the focus is assessing fetal brain anatomy. This study uses data from a scientific research program to characterize the development of functional neural systems beginning *in utero*. The scanning protocol employed in this study prioritized data collection of functional MRI and most functional scanning sessions do not have an anatomical scan. Therefore, the existing structural brain segmentation models are not sufficient for the fetal functional MRI data used in this work. Here, we extend prior work by developing and validating a tool for automatically segmenting the fetal brain from functional MRI data. Ultimately, we connect our auto-masking model with an automated version of a previously manual preprocessing workflow. An overview of the entire suggested preprocessing stream is provided in Fig. 1.

All code discussed in this paper, along with a protocol and a tutorial python notebook, is available on GitHub (https://github.com/saigerutherford/fetal-code). All raw volumes and manually drawn brain masks used for training the convolutional neural network are hosted on OpenNeuro.org (https://openneuro.org/datasets/ds003090). This pipeline addresses the challenges mentioned above and achieves preprocessed fetal time-series data with significantly reduced manual effort. Our work embodies an initial set of guidelines for fetal fMRI preprocessing. The associated protocols are expected to evolve through improvements by the user community in response to new knowledge and innovations in the field.

## Methods

### Participants and Data

Resting-state functional MRI was obtained from two cohorts, Wayne State University (WSU) and Yale University. Eligible participants were at least 18 years of age, assessed as having uncomplicated, singleton pregnancies, and had no contraindications for MRI. WSU cohort consists of 197 fetuses (gestational age 24–39 weeks, M=30.9, SD=4.2). Twenty-one of these fetuses were scanned at two-time points *in utero*. Both time points are included in this study; however, they are counted as a single subject. WSU fetal MR examinations were performed on a Siemens Verio 3T scanner using an abdominal 4-Channel Flex Coil. Scanning protocols were acquired using echo-planar sequence (TR/TE: 2000/30; 4mm slice thickness, axial, interleaved ascending slice order, 360 volumes). Multi-echo resting-state sequences were also collected in a portion of these subjects (TR/TEs: 2000/18,34,50). The Yale University cohort contains ten fetuses scanned twice longitudinally (gestational ages 30-36 weeks, M=32.7, SD=1.9). The Yale scanner was a Siemens Skyra 3T using a 32-channel abdominal coil (TR/TE:2000/30; 3mm slices, 32 slices parallel to the bi-commissural plane, 150 volumes).

Due to the lack of tools for automated segmentation of the fetal brain, research personnel were trained to manually draw fetal brain masks using BrainSuite software (Shattuck & Leahy, 2002). In line with prior work, manually generated brain masks are used to judge the accuracy of automated segmentation methods in the present analysis.

### Auto Masking

#### Experimental Pipeline

Due to multiple manually drawn masks per subject, WSU data were randomly separated at the subject level into training, validation, and test sets with 129, 20, and 48 subjects (855, 102, and 206 volumes), respectively. The training set was used to optimize the model. The validation set was used to gauge the network’s generalization performance during training and determine when to stop training. The test set is held out and used after training was completed to evaluate the model’s performance.

For those interested in using this model on unlabeled data from potentially unseen MRI scanners, we wanted to further demonstrate our model’s generalizability. We re-trained the CNN a second time using the WSU data as training and validation sets (training = 177 subjects/1,066; validation = 20 subjects/102 volumes) and an additional fetal functional dataset (referred to as the Yale test set; 10 subjects/57 volumes) collected in a separate population (Yale University, New Haven, CT) on a different MRI scanner. An overview of the data splitting into single scanner and multi-scanner test sets is shown in Fig. 1A. Minimal preprocessing of the data included resampling and zero-padding the images to consistent voxel sizes (3mm^3^) and dimensions (96 × 96 × 37). Functional time-series data are input to the auto-masking neural network as individual timepoints (i.e., a 3D NIFTI rather than a 4D NIFTI).

#### Network Architecture

The U-Net style CNN network architecture (Falk et al., 2018) implemented in this pipeline was adapted from Salehi et al. (2017, 2018). The architecture features repeated blocks of 3 × 3 convolutions followed by the ReLu activation function assembled into a contracting path, followed by an expanding path. In the contracting path, every second convolution is followed by a 2 × 2 max pooling operation. In the expanding path, every second convolution is followed by a 2 × 2 upsampling process using nearest-neighbor interpolation. Every other feature map in the contracting path is concatenated along the depth dimension to the corresponding map in the expanding path, helping the network learn the appropriate location of the output mask. The final layer is convolved with two 1 × 1 filters to produce an output mask with channels equal to the number of output classes.

The network separates 3D image volumes into 2D axial slices and operates on each slice independently. We chose to implement a 2D rather than 3D network to reduce computational costs. This model includes steps for converting raw NIFTI images into a format readable by the network and steps for converting the network’s output into a NIFTI-formatted, 3D brain mask.

The model was implemented using Tensorflow (version 1.4.1). Training and testing of the network were performed using a GPU, but CPU testing times were also evaluated to provide an additional reference point.

#### Training Procedures

During training, the weights in a CNN are minimized with respect to a loss function that determines how well the network is learning from the training data. We optimized our network via per-pixel cross-entropy, with weights determined using the Adam Optimizer (Kingma & Ba, 2014). Adam is a first-order gradient method that updates the weights adaptively based on previous and current gradients. Even using an adaptive optimizer, we found that using a learning rate decay improved performance. The initial learning rate was set to 0.0001 with an exponential decay rate of 0.9, applied every 10,000 batches. The model was trained until performance no longer improved on the validation set. The 2D axial slices in the training data were augmented through 90-degree rotations and horizontal and vertical flips. While augmentations were done on 2D slices, the same rotations and flips were applied to all slices to preserve the 3D shape. These augmentations capture the non-standard orientation of the brain in fetal volumes.

#### Evaluation

The evaluation process was performed over multiple steps. First, we evaluated our network’s ability to mask the fetal brain using the Dice coefficient. The Dice coefficient measures the percent overlap between two regions: the predicted brain region and the actual brain region. It is defined between zero and one, where zero means there is no overlap between the two areas, and one means the two regions are identical. We also report the Jaccard index, Hausdorff surface distance (Karimi & Salcudean, 2019), sensitivity (true positive rate: brain voxels are correctly identified as brain), and specificity (true negative rate: nonbrain voxels are correctly identified as nonbrain) of our network on the WSU and Yale held-out test sets. Detailed mathematical definitions of these metrics can be found in Taha and Hanbury (2015).

After training, we calculated Dice coefficients for all auto-masks that have a manually drawn counterpart, though we report values only for volumes in the test data, as performance within the train and validation datasets does not reflect model performance on new data and CNNs are known to achieve near perfect performance within the training set.

#### Comparison to Other Methods

Also, to aid in interpretation and benchmarking obtained evaluation metrics, we performed a secondary analysis to demonstrate that current methods for brain extraction perform poorly when applied to fetal data. We used the Brain Extraction Tool (BET) implemented in FSL, 3dSkullstrip from AFNI, and the fetal anatomical U-Net from Salehi et al. (2018), to benchmark our model. Of note, evaluation metrics can be improved by separating testing data into challenging versus non-challenging images (Salehi et al., 2017). This splitting approach is not used here as this diminishes the representativeness of estimates when applied across complex and varied data sets.

#### Failure Analysis

After the model performance was evaluated, we conducted a failure analysis to discover patterns of intrinsic image characteristics that may influence auto-masking performance. First, we examined the relationship between the evaluation metrics and gestational age. Next, we evaluated whether image artifacts, brain size ratio (brain volume relative to the entire image volume), or brain position in the center of imaging space influence model performance by qualitatively examining images with dice coefficients falling below 0.9.

### Application of Auto-masks

Auto-masks are output as spatial probability estimates, wherein voxel values equal to one correspond to the highest probability of being brain. Probability map brain masks were then clustered, thresholded, and binarized. These steps are taken to discard small, non-brain clusters that may have been included in the probability map brain mask. Binarized masks were then resampled back into the subject’s native space and applied to the native image using an image multiplier, resulting in segmented brain volumes corresponding to each fetal fMRI data timepoint.

### Other Preprocessing Steps

In addition to segmentation of the fetal brain from surrounding maternal tissue, fetal imaging preprocessing requires several additional steps: motion denoising, realignment of volumes within a time series, and group-level normalization to a fetal template. These steps are challenging because frame-to-frame displacement is elevated in fetal studies, and the fetal brain is typically not in a single standard orientation.

In prior studies by our group, a reference frame from each quiescent period was chosen to be masked. The mask would then be applied to every volume within the low movement period, not only the volume it was drawn on. Due to the time-consuming nature of manual masking, it was not feasible to mask every volume. Auto-masking’s central goal is to mask an entire time-series in a fraction of the time it takes to manually draw a mask for a single volume. Realigning all volumes within a time series creates parameters (mean framewise displacement) that are used to identify low movement periods that are usable for further activation or connectivity analyses.

Typical realignment of fMRI data is done on full time series using the middle volume (in time) as a reference volume. Due to notably high movement across a fetal time series, using a single reference volume for realignment may not be optimal. In this pipeline, instead of using a 4D NIFTI file in the realignment step, we realign 3D volumes such that a new reference volume is always selected at each iteration. We selected the FLIRT FSL realignment tool (Jenkinson et al., 2002). FLIRT first estimates a linear transformation between volume n and the reference volume (*n + 1*). This step is repeated across all 3D volumes in the time-series and each of these *n* to (*n + 1*) transformation matrices are applied to each volume of the time series to produce a new data set comprised of realigned volumes. This step also creates a text file and plot that summarizes the six rigid-body realignment parameters across time, which can be subsequently used to identify motion outliers and perform motion censoring in later processing stages. Here, we applied the fsl_motion_outliers routine as a data-driven means of defining high and low fetal movement periods.

After masking and realignment, the individual time point 3D volumes are merged back into a 4D NIFTI, moved into group template space using linear warping, and spatially smoothed. Flexibility is built into the pipeline such that the user can define whether data are normalized to a standard reference template or age-specific fetal templates, see Serag et al. (2012). A linear normalization is implemented via FLIRT (Jenkinson et al., 2002). After normalization, all volumes are spatially smoothed with a user-specified Gaussian kernel.

Importantly, all software tools used within this preprocessing pipeline (TensorFlow, Python, AFNI, FSL) are free and open source. All commands can be implemented in a shell script, which can be run from the command line.

### Quality Control

While this methodology employs fully automated techniques for preprocessing fetal resting-state fMRI data, manual quality assurance processes are necessary at crucial transition points throughout the pipeline. Specifically, our standard process includes an initial review of raw time-series data, screened as a movie. Initial inclusion criteria are that the brain is in the field of view and unobstructed by artifacts and that within the time series, there are periods of minimal fetal movement. We exclude data not meeting these criteria. However, most Wayne State University data passes this stage because long scan durations are used, and fetuses rapidly cycle through quiescent states.

Additional steps in the quality control protocol are implemented after auto-masking, realignment, and normalization. At these stages, time-series data are again visually inspected to assure that no errors were introduced during these stages of preprocessing. Several parameters that are automatically generated during the pipeline should be used in complement to manual quality checking. These parameters include realignment parameters, motion plots, and metrics from the fsl_motion_outliers command.

## Results

### Auto-masking Performance

**Fig. 2:**
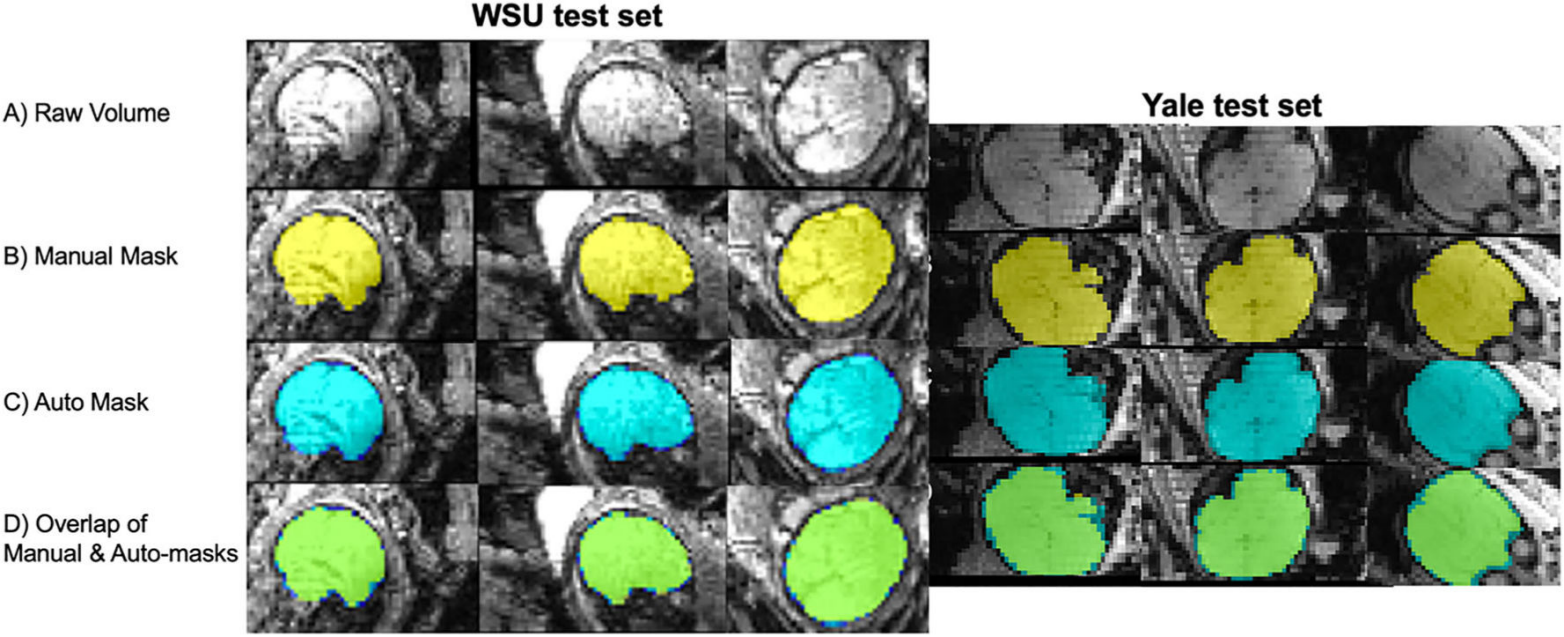
Comparison of manual and automated masks. (A) Raw volume; (B) Hand-drawn mask; (C) Auto mask; (D) Conjunction of hand drawn (yellow) and auto (blue) masks, overlap between hand and auto masks shown in green. *WSU data collected in Detroit, MI, at Wayne State University. Yale data collected in New Haven, CT at Yale University*

Our CNN auto-mask model achieved high accuracy when delineating fetal brain from surrounding structures in fetal brain fMRI images. We evaluated the model on two held-out test sets. Applied to the Wayne State University (WSU – 206 volumes from 48 unique subjects) and Yale (57 volumes from 10 subjects) test cohorts, the models achieved a per-volume average dice coefficient of 0.94 and 0.89, respectively. The CNN’s performance in terms of Dice coefficient, Jaccard coefficient, Hausdorff surface distance, sensitivity, and specificity across both test sets is summarized in Table 1. Figure 2 provides examples of high fidelity between manual and auto-masks in both test sets.

### Comparison to Adult Auto-masking Tools

**Fig. 3:**
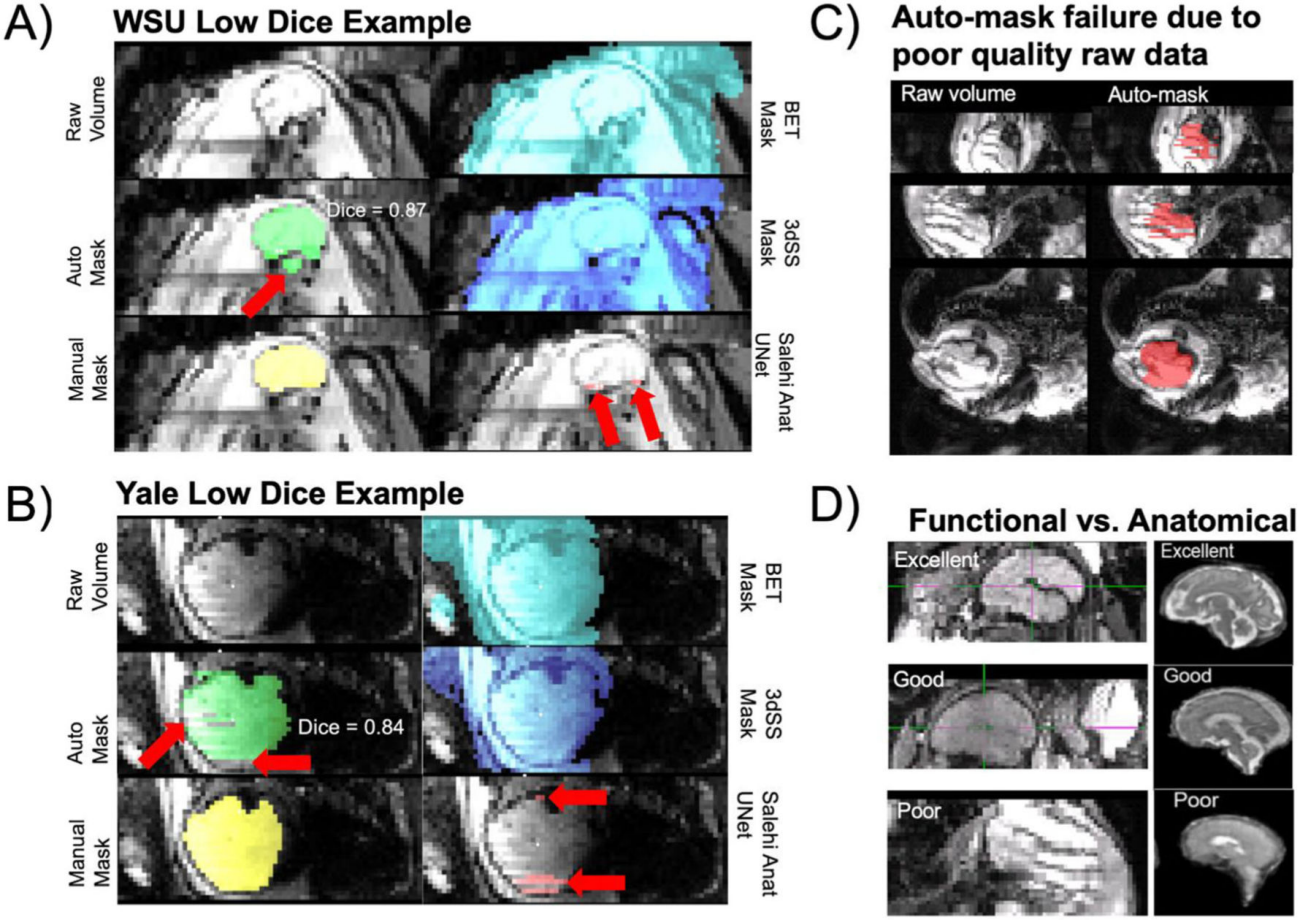
Failure analysis and comparison of functional with structural fetal MRI data. (A) All data of the WSU test set subject with the lowest Dice coefficient (0.87). (B) All data of the Yale test set subject with the lowest Dice coefficient (0.84). The BET, 3dSkullStrip, and Anatomical U-Net masks do not adequately capture the fetal brain’s boundary in both the WSU and Yale case. (C) Extreme failure of the auto-mask model, due to very poor quality of the raw data. (D) Comparison of data quality between fetal functional and structural MRI data to understand why models designed for brain segmentation of anatomical data do not necessarily translate to functional data. Structural fetal MRI image used with permission from Payette et al. (2021)

As a point of reference for the Dice coefficient achieved by our method, we performed an additional analysis using several existing auto-masking tools: Brain Extraction Tool (BET), 3dSkullstrip (3dSS), and the fetal anatomical U-Net from Salehi et al. (2017, 2018). As expected, when applied to the same test set data, these tools performed significantly worse. Evaluation metrics are reported in Table 1, and examples of the masks generated using these tools are shown in Fig. 3. The fetal anatomical U-Net masks were empty in most cases, and therefore the evaluation metrics failed and are not reported in Table 1. In Fig. 2D, functional and structural fetal MRI data are shown side by side to demonstrate the substantial differences in image characteristics between modalities. These results highlight that in fetal functional data, areas of the maternal compartment have high contrast boundaries and varied image intensity creating challenges for standard adult masking routines and severely compromising performance. A network that has been adapted for and trained on fetal functional data is necessary to achieve high-quality functional brain masks.

### Age & Data Quality Failure Analysis

**Fig. 4:**
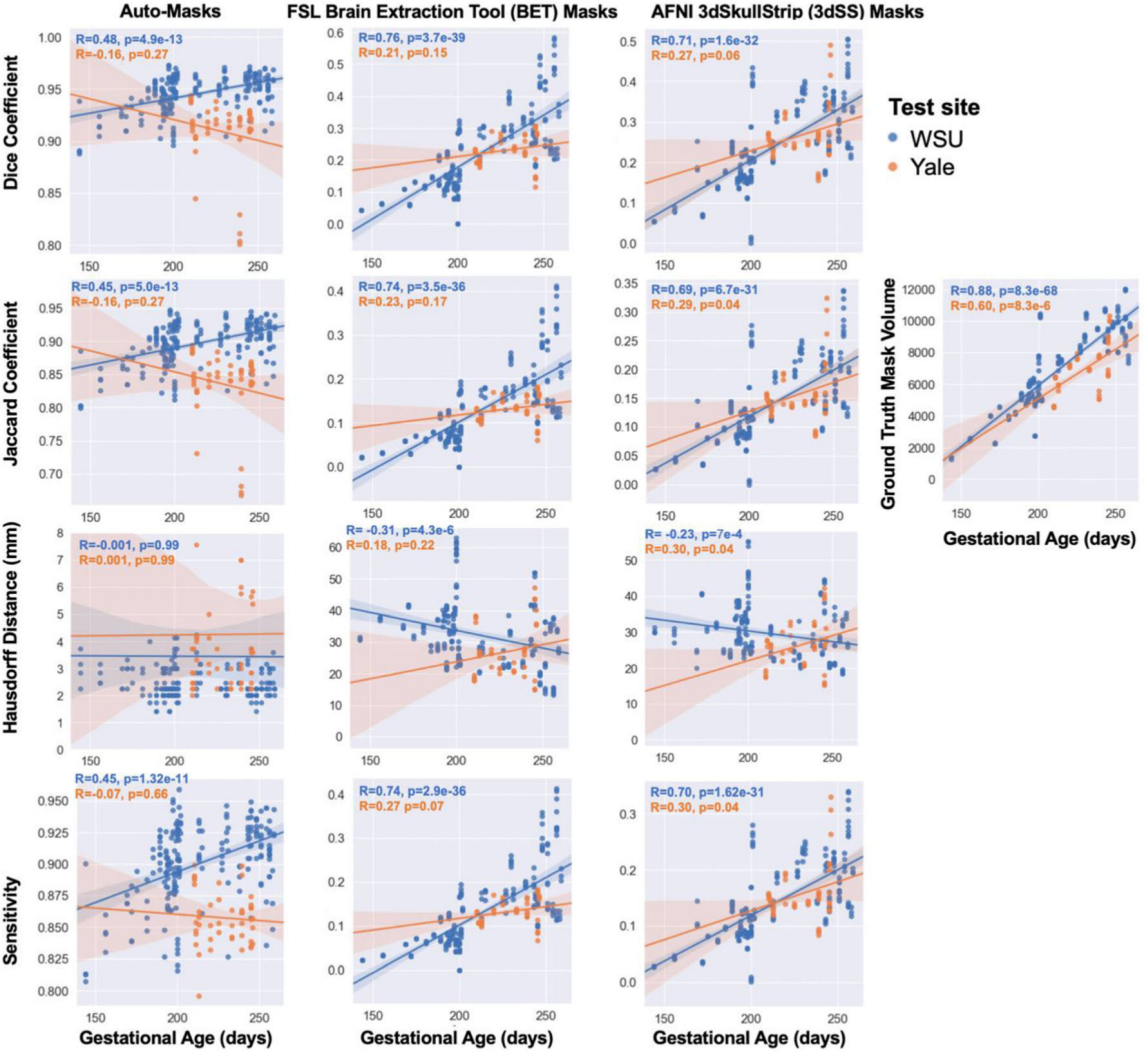
Evaluation of auto-masking model. The relationships between fetal gestational age (in days) at scan are shown on the x-axes and auto-masking performance in the WSU test sets (blue) and Yale test set (orange) on the y-axes. We calculated the evaluation metrics on a per-volume basis, however, the values shown here are on a per-subject basis to examine the relationships with age

Examination of the effect of fetal age on the algorithm’s performance revealed a significant positive association between Dice coefficient and gestational age (Fig. 4). This relationship is likely due to older fetuses having larger brain volumes (Crum et al., 2006). The correlation between gestational age and brain mask volume is highly significant and the correlation between Dice coefficient and brain mask volume is also highly significant. Auto-masks that had “low” Dice coefficients (< 0.9) were visually inspected to further understand reasons for suboptimal performance. Two out of 48 subjects in the WSU test set (4 out of 206 volumes) and two out of ten subjects (6 out of 57 volumes) in the Yale test set had dice coefficients less than 0.9. Examples of the raw and brain mask data from these low Dice subjects are shown in Fig. 3. In this study’s GitHub (https://github.com/saigerutherford/fetal-code), there are video examples of successful and failed auto-masks which better explore the 3-dimensional space. We found that aliasing negatively impacted auto-mask performance (example shown in Fig. 3C) and that the algorithm also performed more poorly for images in which the brain had a large displacement from the image origin.

### Computational Time and Hardware

An often-noted property of deep learning models is their ability to surpass human speed in completing complex tasks substantially. Our auto-masking model illustrates this acceleration. The training time refers to the wall-clock time it took our CNN model to converge to a set of weights that minimize the Dice coefficient on the validation set. The training was stopped after signs of overfitting were observed, that is, performance on the validation set was no longer increasing. The model’s total training time was 3 h and 46 min on a GeForce GTX 1080 Ti GPU. Testing time refers to the time it takes to run the CreateMask.py script to load the raw input volume (NIFTI to NumPy array conversion) and output a predicted auto-mask (as a NIFTI file). The testing time to generate a new auto brain mask is approximately 0.2 s if using a GPU, and 2.5 s if using CPU.

### Other Preprocessing

An additional benefit of our approach for auto-masking all individual volumes is that multiple realignment strategies are now possible. After the fetal brain has been extracted, the time series data can enter more typical preprocessing steps for which child/adult tools have been developed. The main difference when applying these tools is that the fetal brain can have any orientation (meaning image origins may be very far apart), and fetal data exhibits substantially increased head motion.

**Fig. 5:**
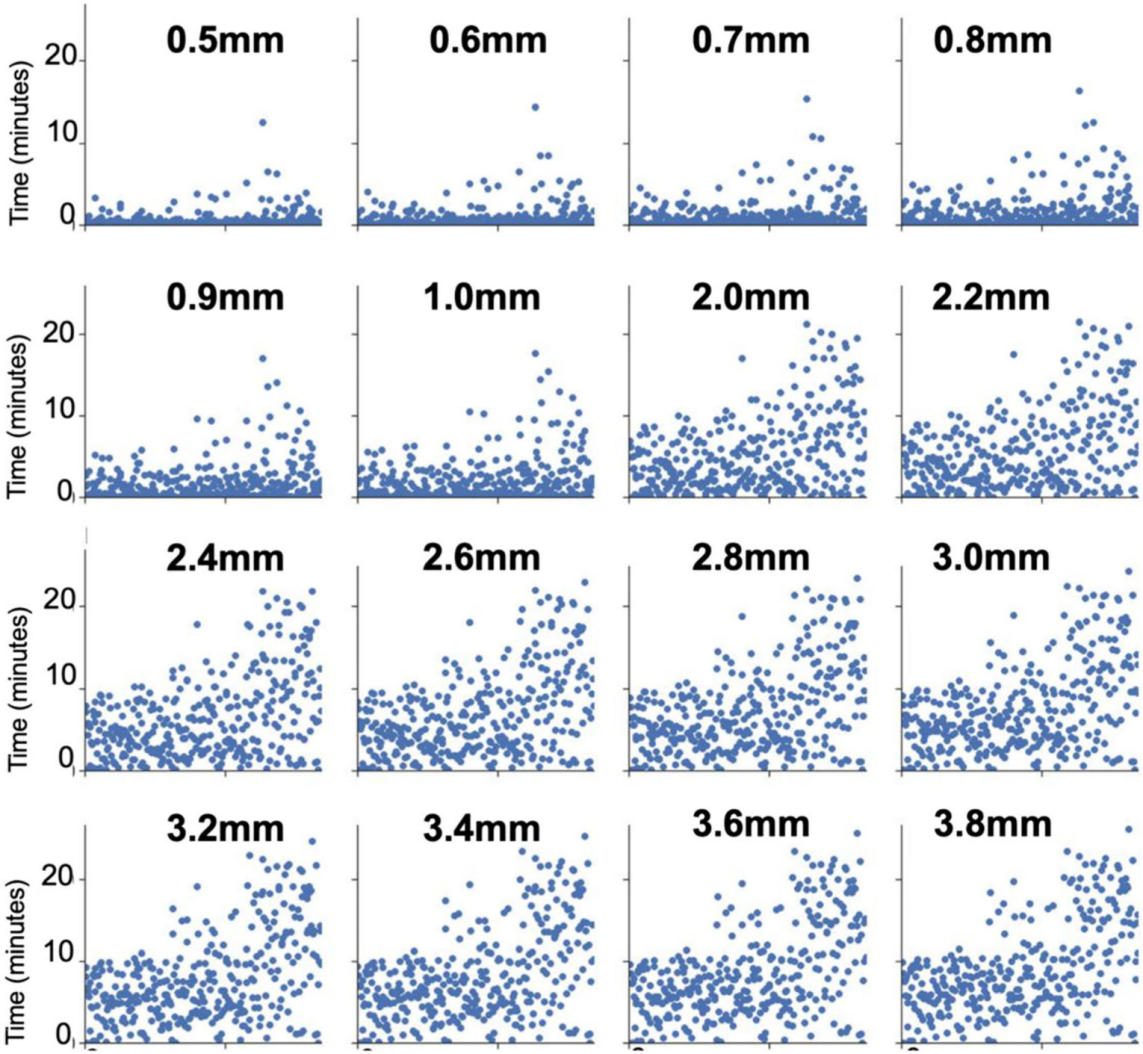
Motion summary. Framewise displacement (FD) censoring thresholds from 0.5mm – 3.8mm were tested, and the amount of data remaining for each subject is shown at each threshold. Each subplot represents a different FD threshold (bolded above the subplot). Each subject represents a point on the x-axis, and the y-axis shows the time, in minutes, remaining after removing high movement volumes

Concerning the problem of elevated head motion, errors introduced by movement cannot, at present, be fully corrected in the fMRI time series. This fact necessitates the application of stringent criteria for retaining only low-motion volumes to ensure data integrity, which is the approach taken by most studies to date (Jakab et al., 2014, 2015; Schöpf et al., 2012; Thomason et al., 2017; van den Heuvel et al., 2018). The correct framewise displacement threshold to censor high motion volumes from a BOLD time-series is an active discussion topic. The answer to the framewise displacement threshold debate is complex, and a concrete solution is outside the scope of this work. However, we tested many different censoring thresholds, and the resulting amounts of data preserved at each censoring threshold are shown in Fig. 5.

## Discussion

Fetal functional MRI is an emerging field with great potential to improve understanding of human brain development. The number of papers published in this area has seen a 5-fold increase since 2010. However, methodologies for processing these complex data sets have not kept pace, and the absence of a standard publicly available processing pipeline for these data has been especially notable. Here we address this gap and present a solution to the most cumbersome processing step, localization, and extraction of the fetal brain from surrounding tissue for each volume of a functional time series. This is a necessary step in processing human fetal fMRI data. Until now, it has been a rate-limiting factor in accurate, automated processing of fetal fMRI BOLD time-series data. This model was built by pairing a set of 1,241 hand-drawn fetal 3D brain masks with a deep learning algorithm, a U-Net convolutional neural network. Pipeline code and documentation are made available through GitHub (https://github.com/saigerutherford/fetal-code). Training, validation, and test data sets (raw volumes, hand-drawn masks, and auto-masks) are available in BIDS format on OpenNeuro.org (https://openneuro.org/datasets/ds003090). It is hoped that the release of an easy-to-use, efficient, validated auto-masking pipeline will reduce barriers for new labs to enter this area while also providing experienced labs opportunities for further optimization of their independently developed approaches. Future integration of this auto-masking model will be incorporated into BioImage Suite (https://bioimagesuiteweb.github.io/webapp/), a web-based image analysis tool that will make use of this model even more accessible to the field.

Recent pioneering work has established that deep learning approaches are effective in fetal *structural* brain segmentation (Ebner et al., 2020; Ison et al., 2012; Khalili et al., 2019; Klinder et al., 2015; Link et al., 2017; Makropoulos et al., n.d.; Payette et al., 2021; Rajchl et al., 2016; Salehi et al., 2017, 2018; Serag et al., 2017; Tourbier et al., 2017). However, fetal *functional* imaging presents a distinct set of constraints and therefore requires a different solution. In particular, the inherently lower resolution and contrast of functional time-series data, and the 4-dimensional nature of the data (~ 360 3D volumes per subject), make this a more challenging problem. Here we applied CNN methods to the most extensive fetal fMRI data set reported to date, 207 fetuses, and derived a novel method for accurate and reliable segmentation of the fetal brain from surrounding maternal tissues within a fraction of a second, 94% accuracy in 0.2 s. These encouraging results are partially attributable to the large set of manually traced human fetal fMRI masks used to train the CNN, consistent with studies showing the correlation between CNN performance and the training data size (Cho et al., 2015; Verghese et al., 2018).

The auto-masking model exhibited strong generalizability, which is particularly important given known biases in CNN models trained on imaging datasets from a single site/population (Tommasi et al., 2015; Zech et al., 2018). First, the trained CNN correctly classified data at two held out sets with 94 and 89%, very similar to the training data. This finding suggests that the CNN is robust to variations in experimental procedures, scanner settings, and populations studied. Also, the training images were drawn from a wide fetal age-range, which should also enhance generalizability across fetal samples encompassing different ages.

Our aim in this project goes beyond brain segmentation; we sought to construct an automated version of a previously manual preprocessing pipeline that is standardized but flexible and readily deployable across multiple data sources. Thus, our pipeline begins with an auto-masking step, leverages existing algorithms (FSL, AFNI) that assist with applying the auto-masks, performs frame-to-frame alignment, normalization to a user-defined template, and finally smoothing. The user is referred to publicly available multi-age fetal brain templates (Serag et al., 2012) and can easily configure the tool to modify or eliminate steps. Our code’s flexibility allows for potential users to mix and match the portions of this pipeline they wish to use. For example, a user could choose an alternative realignment algorithm as the first step then apply the auto-masking step. The tool’s open construction will allow the incorporation of future processing advances, such as surface-based registration and additive motion correction strategies.

Our work has several limitations. First, deep learning methods perform classification in high dimensional space, and consequently, results can be a “black box” with little opportunity for interpretation of axes (Cabitza et al., 2017). However, this limitation should be viewed in the context of our goal: to automatically perform a task that can take trained individuals many hours to perform manually. We are less interested in understanding computer-based brain masking mechanisms and instead focus on algorithm performance in out of sample data sets.

Another limitation is that we use direct warping, or normalization, of functional data to a group-averaged anatomical template. It is not clear that alternatives would improve registration significantly, but one might expect registration to subject-specific anatomy, then to template space, to be a preferred approach. This approach’s challenge is that obtaining high-quality subject-specific high-resolution anatomical images presents a different set of challenges that have been addressed elsewhere (Studholme, 2015). Prior studies of fetal anatomical development (Nunes et al., 2018) demonstrate that even when trained experts apply the most advanced techniques to these data, there is still significant data loss and image blurring where motion effects, image artifacts, or lack of tissue contrast compromise data quality. This example extends to other parts of the pipeline, where alternative optimized preprocessing strategies could be used. However, this work’s objective is not to serve as a final fetal functional MRI preprocessing endpoint but as a backbone upon which further development can follow.

A final limitation is that ours is not a fully automated pipeline. It requires human supervision and quality checking at several stages, which requires a certain quantity of time and level of expertise from the human supervisor. Fortunately, however, the level of involvement and associated expertise required is limited and includes looking for overt errors when viewing processed images as a continuous movie, which takes approximately 1–5 min per functional run. Assuming the entire time for running this pipeline is 15–30 min, including human effort, this is a 60-fold time reduction over prior methods, with manual tracing in particular requiring extensive time and substantial expertise (Thomason et al., 2017; van den Heuvel et al., 2018).

There are many future directions to continue improving fetal functional MRI preprocessing and data analysis. Regarding the auto-masking portion of the pipeline, future contributors to this pipeline should consider using 3-D instead of 2-D convolutions and directly using the probability masks (instead of binarizing them) to utilize the model’s uncertainty estimates. Regarding the realignment and quality checking portions, it may be useful to calculate the Dice coefficient between consecutive volumes (after realignment) as a metric of data quality – a dice coefficient equal to one represents two perfectly images. Other advanced alignment algorithms to register the data to template space should also be investigated and adapted for fetal data (Bozek et al., 2018; Robinson et al., 2014, 2018). It is important to benchmark new brain segmentation models against existing solutions. In order for benchmarking to become common practice, pre-trained models must be shared along with the code. There are expensive computational costs to re-training a model every time a new user wants to use it, and these costs can be eliminated by sharing pre-trained model weights. Furthermore, the code to test existing brain segmentation models must be accompanied by clear directions for setup and testing, and ideally a tutorial showing step-by-step how to successfully run the code. We encourage users who test the auto-mask pipeline introduced in this paper in their fetal data sets to report performance metrics back to the study’s GitHub page. Also, if new users re-train the auto-mask model by adding additional labeled data from their site, it would be beneficial to the fetal imaging community to share these pre-trained models on our study’s GitHub (via a pull request to our repository). A contributing guide on the GitHub page provides instructions for sharing additional pre-trained models. This will ensure that new users are able to access the most current, best performing auto-masking model and will lower the barriers to entry for fetal functional imaging. Finally, it would be beneficial to follow in the footsteps of an extremely successful adult MRI preprocessing pipeline, fMRIPrep (Esteban et al., 2018), and package the code into a container, either Docker (Merkel, 2014) or Singularity (Kurtzer, 2016). A container is a standard unit of software that packages up code and all its dependencies, so the application runs quickly and reliably from one computing environment to another (What Is a Container?, n.d.).

Large-scale, often multi-center, projects are becoming the new norm, and these require validated, standardized processing pipelines of the kind that we have developed in this work. The Developing Human Connectome Project provides just one example of a large-scale study that includes a fetal functional MRI component (Bozek et al., 2018; Fitzgibbon et al., 2020; Harms et al., 2018; Makropoulos et al., n.d.) and many more large-scale fetal fMRI initiatives will likely emerge in the coming years.

In sum, in this work, we leverage deep learning methods in the most significant sample of fetal fMRI data published to date to address the challenging brain segmentation problem in fetal fMRI. We unite our novel auto-masking tool with other preprocessing steps to initialize the first complete open-source solution to preprocessing raw fetal functional MRI time-series data.

## Figures and Tables

**Table 1:** Performance of auto-mask model and existing masking software evaluated in two independent test sets from Wayne State University (WSU) and Yale University. Values reported are the mean (s.d.) within the test sets

	WSU Auto-mask	WSU BET	WSU 3dSS	Yale Auto-mask	Yale BET	Yale 3dSS
Dice	0.94 (+/− 0.067)	0.22 (+/− 0.13)	0.24 (+/− 0.10)	0.89 (+/− 0.13)	0.22 (+/− 0.06)	0.25 (+/− 0.08)
Jaccard	0.89 (+/− 0.069)	0.13 (+/− 0.086)	0.14 (+/− 0.07)	0.82(+/− 0.13)	0.13 (+/− 0.03)	0.15 (+/− 0.05)
Hausdorff Distance (mm)	12.11 (+/− 22.4)	112.6 (+/− 36.7)	103.3 (+/− 26.0)	19.25 (+/− 14.5)	95.2 (+/− 30.1)	92.8 (+/− 24.2)
Sensitivity	0.90 (+/− 0.04)	0.13 (+/− 0.08)	0.14 (+/− 0.07)	0.84 (+/− 0.12)	0.12 (+/− 0.03)	0.15 (+/− 0.05)
Specificity	0.99 (+/− 0.0007)	0.99 (+/− 0.003)	0.99 (+/− 0.002)	0.99 (+/− 0.002)	0.99 (+/− 0.004)	0.99 (+/− 0.002)

Auto-mask is our proposed model. BET is Brain Extraction tool from FSL, 3dSS is 3dSkullStrip tool from AFNI
